# Concurrent Detection of Cognitive Impairment and Amyloid‐Beta PET Status with a Combination of Digital Clock and Recall and Digital Trail Making Test‐Part B

**DOI:** 10.1002/alz70856_107677

**Published:** 2026-01-09

**Authors:** Ali Jannati, Karl Thompson, Claudio Toro‐Serey, Connor Higgins, David Bates, Alvaro Pascual‐Leone, Sean Tobyne

**Affiliations:** ^1^ Linus Health, Boston, MA, USA; ^2^ Hebrew SeniorLife, Boston, MA, USA; ^3^ Harvard Medical School, Boston, MA, USA

## Abstract

**Background:**

The efficacy of disease‐modifying treatments (DMTs) for Alzheimer's disease (AD) hinges on early detection of cognitive impairment and abnormal brain amyloid‐beta (Aβ) levels. Moreover, AD clinical trials face significant barriers of high screening failure rates and expensive prescreening. Therefore, there is an urgent need to address these challenges by developing a more efficient and cost‐effective method for early AD identification and differentiation from other etiologies. This study evaluated the performance of a combination of Linus Health Digital Clock and Recall (DCR), a 3‐minute digital cognitive assessment, and digital Trail‐Making Test‐Part B (dTMT‐B) in detecting cognitive impairment and predicting brain Aβ‐PET status.

**Method:**

930 participants (mean age 72.0±6.7; 56.8% female; 23% minorities) in the Bio‐Hermes‐001 study were classified as cognitively unimpaired (CU), mild cognitive impairment (MCI), or probable Alzheimer's dementia (pAD), and 35.1% were Aβ+ on 18F‐florbetapir PET scan. A 3‐class, cross‐validated machine‐learning ensemble model combined multimodal process‐based features of DCR and dTMT‐B including drawing metrics, temporal‐spatial features of stylus manipulation, speech and acoustic features, demographics, and APOE status. All models had <22% indeterminate (Ind.) cases.

**Result:**

The DCR+dTMT‐B model had AUC=0.906 (NPV=0.816; PPV=0.900) for differentiating HC vs. MCI/pAD vs. Ind., AUC=0.891 (NPV=0.872; PPV=0.808) for differentiating HC vs. MCI vs. Ind., AUC=0.950 (NPV=0.941; PPV=0.870) for differentiating HC vs. pAD vs. Ind., and AUC=0.920 for differentiating MCI vs. pAD vs. Ind. (NPV=0.886; PPV=0.840). For predicting Aβ‐PET status, the DCR+dTMT‐B model had AUC=0.890 (NPV=0.914; PPV=0.754), whereas the DCR+dTMT‐B+APOE model achieved AUC=0.933 (NPV=0.933; PPV=0.815).

**Conclusion:**

A multimodal model combining process‐based features of DCR and dTMT‐B had very good to excellent performance in cognitive‐impairment detection and excellent performance in Aβ‐PET status prediction, with the DCR+dTMT‐B+APOE model performing comparably to CSF biomarkers of AD. This model enables prioritizing the most suitable patients for BBM testing and DMTs, and substantially increases the efficiency of recruitment for AD clinical trials.

